# The Citrus Flavonoid Naringenin Protects the Myocardium from Ageing-Dependent Dysfunction: Potential Role of SIRT1

**DOI:** 10.1155/2020/4650207

**Published:** 2020-01-25

**Authors:** Lara Testai, Eugenia Piragine, Ilaria Piano, Lorenzo Flori, Eleonora Da Pozzo, Vincenzo Miragliotta, Andrea Pirone, Valentina Citi, Lorenzo Di Cesare Mannelli, Simone Brogi, Sara Carpi, Alma Martelli, Paola Nieri, Claudia Martini, Carla Ghelardini, Claudia Gargini, Vincenzo Calderone

**Affiliations:** ^1^Department of Pharmacy, University of Pisa, Via Bonanno 6, 56126 Pisa, Italy; ^2^Interdepartmental Research Center “Nutraceuticals and Food for Health”, University of Pisa, Via del Borghetto 80, 56124 Pisa, Italy; ^3^Interdepartmental Research Center of Ageing, Biology and Pathology, University of Pisa, Via Savi 10, 56126 Pisa, Italy; ^4^Department of Veterinary Sciences, University of Pisa, Viale delle Piagge 2, 56124 Pisa, Italy; ^5^Department of Neuroscience, Psychology, Drug Research and Child Health-NEUROFARBA-Section of Pharmacology and Toxicology, University of Firenze, Via Gaetano Pieraccini 6, 50139 Firenze, Italy

## Abstract

Sirtuin 1 (SIRT1) enzyme plays a pivotal role in the regulation of many physiological functions. In particular, it is implicated in ageing-related diseases, such as cardiac hypertrophy, myocardial infarct, and endothelial dysfunction; moreover, its expression decreases with age. Therefore, an effective strategy to extend the lifespan and improve cardiovascular function is the enhancement of the expression/activity of SIRT1 with exogenous agents. The Citrus flavonoid naringenin (NAR) presents structural similarity with the natural SIRT1 activator resveratrol. In this study, we demonstrate through *in vitro* assays that NAR significantly activates SIRT1 enzyme and shows antisenescence effects. The binding mode of NAR into SIRT1 was detailed investigated through *in silico* studies. Moreover, chronic administration (for six months) of NAR (100 mg/kg/day) to 6-month-old mice leads to an enhancement of SIRT1 expression and a marked reduction of reactive oxygen species production in myocardial tissue. Furthermore, at the end of the treatment, the plasma levels of two well-known markers of cardiovascular inflammation, TNF-*α* and IL6, are significantly reduced in 12-month-old mice treated with NAR, as well as the cardiovascular risk (total cholesterol/HDL ratio) compared to control mice. Finally, the age-associated fibrotic remodeling, which is well detected through a Mallory trichrome staining in the vehicle-treated 12-month-old mice, is significantly reduced by the chronic treatment with NAR. Moreover, an improvement of myocardium functionality is highlighted by the enhancement of citrate synthase activity and stabilization of the mitochondrial membrane potential after NAR treatment. Taken together, these results suggest that a nutraceutical approach with NAR may have positive impacts on many critical hallmarks of myocardial senescence, contributing to improve the cardiac performance in aged subjects.

## 1. Introduction

Ageing is a physiological process associated with a progressive impairment of homeostasis at cellular, tissue, and systemic level that increases the susceptibility to several ageing-related diseases. In this context, the heart is one of the main organs at risk; indeed, myocardial remodeling, accompanied by collagen fiber deposition, is considered the gateway to severe cardiac diseases, such as fibrosis and myocardial hypertrophy and heart failure [1].

In this scenario, oxidative stress plays a critical role. Since mitochondria are the main source of reactive oxygen species (ROS), the oxidative stress condition, typical of ageing, can be considered as a consequence of the decline of the mitochondrial bioenergetics. On the other hand, the high age-related production of ROS is also responsible for worsening of its own functionality [2]. Moreover, an age-related low-grade chronic inflammation, defined inflammaging, has been described [3]. In this regard, macrophages are a key source of proinflammatory molecules, such as tumor necrosis factor- (TNF-) *α*, interleukin (IL) 6, and matrix metalloproteinases (MMPs), responsible in turn for the persistence of the basal inflammatory response [4].

Moreover, also sirtuin 1 (SIRT1) enzyme, a type III histone deacetylase able to regulate a plethora of intracellular signaling proteins, is implicated in ageing-related diseases, such as cardiac hypertrophy, myocardial infarct, and endothelial dysfunction. Noteworthly, SIRT1 protects against endothelial dysfunction [5, 6] and slows down the progression of cardiovascular senescence [7]. Furthermore, its expression/activity is inversely related with ischemia-reperfusion damage or with severity of heart failure [8, 9].

The expression of SIRT1 enzyme gradually decreases with age, and SIRT1-deficient mice show a reduced lifespan [10].

Therefore, considering that ageing is a complex multifactorial process and the maintenance of the cell homeostasis is relevant for the preservation of a healthy state, many efforts are being made to identify natural and synthetic compounds effective on these potential targets.

Naringenin (NAR), a flavanone typical of Citrus fruits, seems to be a promising candidate. Indeed, previous studies demonstrated its beneficial cardiovascular activities, both as vasorelaxing and cardioprotective agent [11–14]. Moreover, we recently observed that NAR also exerts antisenescence effects. In particular, in H9c2 cardiomyoblasts submitted to oxidative stress by H_2_O_2_ treatment, NAR contained the *β*-galactosidase activity, as well as the expression of p21 and p16, well-known markers of ageing [15]; furthermore, bergamot juice, containing a high amount of NAR, increased the genic expression of the antiageing markers SIRT1, Nrf2, and FOXO1 in cardiac tissues [16]. Noteworthly, NAR exhibits close structural analogies with well-known SIRT1 activators, such as such as resveratrol (RES) and quercetin [17–20]. Therefore, we hypothesize that NAR can possess an interesting antisenescence SIRT1-mediated profile, useful as nutraceutical tool in humans. In this work, we investigated the effects of chronic administration of NAR on myocardial ageing using an *in vivo* approach in mice.

## 2. Materials and Methods

### 2.1. Activation of SIRT1 *In Vitro*

The effects of NAR (purchased as pure compound from Sigma-Aldrich, USA) 10, 30, 100, and 300 *μ*M on SIRT1 activity were evaluated by a direct enzymatic assay using a SIRT1 Direct Fluorescent Screening Assay Kit (Cayman Chemical, USA), following the protocol user guide. Fluorescence was analyzed with the EnSpire spectrofluorometer (PerkinElmer, USA) at a wavelength of 350-360 nm in excitation and 450-465 nm in emission. The increase in recorded fluorescence was directly proportional to the activation of SIRT1.

*Data analysis*. Data obtained were analyzed by removing baseline and normalizing to the fluorescence value of the vehicle (0%) and the SIRT1 activator resveratrol (RES, Sigma-Aldrich, USA) 100 *μ*M (100%). One-way ANOVA followed by Bonferroni's post hoc test was selected as statistical analysis, and the difference between groups was considered statistically different when *p* < 0.05.

### 2.2. Computational Study about the Activation of SIRT1 by NAR

The calculation was performed on a system comprising 56 Intel Xeon E5-2660 v4@2.00 GHz processors and two NVIDIA GeForce RTX 2070 GPU, running Maestro Molecular Modeling environment release 2018 (Schrödinger, LLC, New York, NY, 2018).

#### 2.2.1. Computational Details

*Molecule preparation*. The three-dimensional structures of the NAR and RES were downloaded by PubChem and imported into Maestro. Subsequently, energy minimization of conformers was achieved by means of MacroModel software (MacroModel, Schrödinger, LLC, New York, NY, 2018) as reported by us [21, 22], employing the OPLS-AA 2005 as force field and GB/SA model for simulating the solvent effects. PRCG method with 1000 maximum iterations and 0.001 gradient convergence threshold was employed. Furthermore, LigPrep (LigPrep, Schrödinger, LLC, New York, NY, 2018) was employed in order to refine the chemical structures.

*Protein preparation*. SIRT1 three-dimensional structure in complex with RES was downloaded from Protein Data Bank (PDB) (PDB ID: 5BTR [23]) and submitted to the protein preparation wizard protocol implemented in Maestro suite 2018 (Protein Preparation Wizard workflow 2018) in order to obtain a reasonable starting structure for performing *in silico* studies [21, 24].

*Molecular docking*. Docking experiments were performed by Glide (Schrödinger, LLC, New York, NY, 2018) using the ligands and the protein prepared as mentioned above employing Glide extra precision (XP) method as scoring function. The energy grids were prepared using the default value of the protein atom scaling factor (1.0 Å) within a cubic box centered on the crystallized RES molecules. After grid generation, the ligands (NAR and RES) were docked into the enzymes considering the three different potential binding sites of RES. The number of poses entered to postdocking minimization was set to 1000, and the Glide XP score was evaluated. The XP method was able to correctly accommodate the RES into the three binding sites (data not shown).

### 2.3. *In Vitro* Antisenescence Activity

#### 2.3.1. Cell Culture

H9c2 cells are commonly used as a successful cellular model for studies of myocardial ageing [13, 15, 16]. H9c2 cells (ATTC, USA) were cultured in DMEM high glucose, supplemented with 10% FBS, 100 units/mL penicillin, and 100 mg/mL streptomycin in tissue culture flasks at 37°C in a humidified atmosphere of 5% CO_2_.

#### 2.3.2. Senescence-Associated *β*-Galactosidase Staining

H9c2 cardiomyocytic cells were treated with hydrogen peroxide (H_2_O_2_) in order to induce cellular senescence, as previously reported [15, 16, 25, 26]. Cells were seeded onto 24-well plates (10 × 10^3^ cells/cm^2^), and after 24 h, cells received H_2_O_2_ (60 *μ*M) treatment or relative vehicle (distilled water) for 3 h. Then, the medium was replaced with fresh medium containing NAR, 40 *μ*M, RES (25 *μ*M), sirtinol, a selective SIRT 1 blocker (SIR, 20 *μ*M), alone or in combination with NAR or RES, and relative control (0.01% DMSO). After 72 hours, the sa-*β*-Gal staining was assessed [16], fixing the cells in p-formaldehyde and incubating them in staining solution for 16 h at 37°C. Following phosphate-buffered saline washing, images of cells by light microscopy were captured (10x magnification, 5 random fields per well), and both blue and total cells were counted using ImageJ (ImageJ Software, version 1.41, USA).

### 2.4. *In Vivo* Chronic Treatment

Male six-month-old C57BL/6J mice (ENVIGO, Milan, Italy) were randomly assigned into two different groups: one was daily treated with NAR (100 mg/kg) diluted in water while the other one was treated with vehicle (DMSO 1%) up to twelve months of age. Untreated three-month-old mice were used as young controls. The diluted solutions were daily prepared; moreover, mice were weekly weighted, and water intake was daily monitored during the six-month treatment. The dosage of NAR (100 mg/kg) has been selected based on previous *in vivo* experiments [13, 27] and in accordance with papers of others [28–31]. In particular, NAR has been dissolved in DMSO 40 mg/mL and diluted in water up to 0.4 mg/mL, to assure a daily dosage of 100 mg/kg.

All procedures were performed according to European (EEC Directive 2010/63) and Italian (D.L. 4 March 2014 n.26) legislation. Experiments have been reported according to the ARRIVE guidelines [32]. Animals were housed in cages with free access to standard food pellets and water under standard conditions (12-hour light/dark cycle, 22°C). At the end of treatment, mice were anesthetized with an aqueous solution of urethane (30% *w*/*w*), i.p. (Sigma-Aldrich, USA); then, blood was collected and the heart explanted to functional, histological, and biochemical analysis.

#### 2.4.1. Measurement of Lipid and Glucose Levels in Blood

At the end of the treatment, blood was collected from the tail tip of each mouse and fasting blood glucose levels were determined using Glucocard™ blood glucose meter (Menarini, Italy). Then, animals were anaesthetized with an intraperitoneal injection of aqueous urethane solution 30% *w*/*w*. Intracardiac blood was collected in tubes with the anticoagulant EDTA (BD Vacutainer), and complete lipid panel (triglycerides, total cholesterol, HDL, and LDL) and glycated haemoglobin levels were measured with Cobas b 101 instrument (Roche Diagnostics, Switzerland).

*Data analysis*. Blood samples were collected from 15 mice per group and results were expressed as mean ± SEM. Cardiovascular risk was expressed as ratio between total cholesterol and HDL cholesterol levels. One-way ANOVA followed by Bonferroni's post hoc test was selected as statistical analysis, and the difference between groups was considered statistically different when *p* < 0.05.

#### 2.4.2. Cardiac Mitochondria Isolation

Hearts were explanted and immediately put in an ice-cold isolation buffer (composition: sucrose 250 mM, Tris 5 mM, EGTA 1 mM, pH 7.4). The ventricular tissue has been rapidly cut and homogenized with an Ultra-Turrax homogenizer (IKA-Werke GmbH & Co., Germany); three homogenization cycles on ice were performed. Then, the suspension was centrifuged at 1075×g for 3 minutes at 4°C (EuroClone, Speed Master 14 R centrifuge, Italy), and the resulting supernatant was centrifuged at 11950×g for 10 minutes at 4°C. The pellet was resuspended in an isolation buffer without EGTA and centrifuged at 11950×g for 10 minutes at 4°C. Finally, the mitochondrial pellet was suspended in the isolation buffer without EGTA and stored on ice during the experiment. Mitochondrial protein concentration was determined by Bradford assay. All reagents were purchased from Sigma-Aldrich, USA.

*Measurement of Mitochondrial Membrane Potential (ΔΨ)*. Mitochondrial membrane potential was evaluated with a potentiometric method, using a tetraphenylphosphonium- (TPP^+^-) sensitive minielectrode and a reference electrode (WPI, USA), both connected with a data acquisition software (Biopac Inc., USA). Firstly, known concentrations of TPP^+^Cl^−^ (Sigma-Aldrich, USA) were used to calibrate the electrodes. Then, mitochondria (1 mg protein/mL) were added to the incubation buffer (composition: KCl 120 mM, K_2_HPO_4_ 5 mM, Hepes 10 mM, succinic acid 10 mM, MgCl_2_ 2 mM, TPP^+^Cl^−^ 10 *μ*M, pH 7.4), and they were continuously stirred with a small magnet to generate a mitochondrial suspension.

*Data analysis*. Mitochondrial membrane potential was calculated by a modified Nernst experimental equation (1):

(1) $$\begin{array}{c} \Delta \Psi =60x\log\frac{V_{0}\cdot \left({\left[\text{TPP}\right]}^{+}/{\left[\text{TPP}\right]}^{+}\right)-V_{t}-K_{0}P}{V_{m}P+K_{i}P}, \end{array}$$

where ΔΨ is the mitochondrial membrane potential (mV), *V*_0_ is the volume of the incubation buffer before the addition of mitochondria, *V*_*t*_ is the volume of the incubation buffer after the addition of mitochondria, *V*_*m*_ is the volume of mitochondrial matrix (taken as 1 *μ*L/mg of proteins), TPP^+^_0_ is the concentration of TPP^+^ recorded before the addition of mitochondria while TPP^+^_*t*_ is the concentration of TPP^+^ recorded at time *t*, *K*_0_ and *K*_*i*_ are apparent external and internal partition coefficients of TPP^+^ (14.3 *μ*L/mg and 7.9 *μ*L/mg, respectively), and *P* is the protein concentration (expressed in mg).

The mitochondrial membrane potential was evaluated on 6 different animals per group, and data were expressed as mean ± SEM. One-way ANOVA followed by Bonferroni's post hoc test was used to compare groups for statistical differences (*p* < 0.05).

*Measurement of Mitochondrial Citrate Synthase Activity*. Mitochondria were diluted in Tris buffer 100 mM (pH 8.2) containing 5,5′-dithiobis-(2-nitrobenzoic) acid (DTNB, 100 *μ*M) and Acetyl Coenzyme A (100 *μ*M); then, they were treated with Triton X-100 (0,02% *v*/*v*) for 5′ at room temperature to lyse mitochondrial membranes, thus releasing citrate synthase enzyme. The assay was performed in multiwell plates, and the reaction was initiated by the addition of oxalacetate 500 *μ*M. Absorption was measured spectrophotometrically at 30°C and 412 nm every 15 seconds for 3 minutes using a microplate reader (EnSpire, PerkinElmer, USA). All reagents were purchased from Sigma-Aldrich, USA.

*Data analysis*. Mitochondrial citrate synthase activity was determined by comparison to a calibration curve obtained with known concentrations of the enzyme. Cardiac mitochondria were isolated from the hearts of 8 animals per group, and they were expressed as mean ± SEM. One-way ANOVA followed by Bonferroni's post hoc test was selected as statistical analysis, and the difference between groups was considered statistically different when *p* < 0.05.

#### 2.4.3. Western Blot Analysis of SIRT1 Enzyme

A portion of the left ventricular tissue was lysed in RIPA buffer, and proteins were quantified with the Bradford assay (Bio-Rad, USA). Proteins (25 *μ*g) were separated onto a precast 4-20% polyacrylamide gel (Mini-PROTEAN® TGX gel, Bio-Rad, USA) and transferred to PVDF membranes (Trans-Blot® Turbo™ PVDF Transfer packs, Bio-Rad, USA). Membranes were blocked with 5% bovine serum albumin (BSA) diluted in Tris-buffered saline (TBS, 20 mM Tris-HCl, pH 7.5, 150 mM NaCl) with 0.1% Tween 20. Primary antibodies against GAPDH (1 : 5000, Millipore) and SIRT1 (1 : 100, Santa Cruz, USA) were incubated overnight at 4°C. Then, membranes were incubated with secondary antibodies (1 : 5000, anti-mouse and anti-rabbit) for 2 h at room temperature. The immunoblot signal was visualized by using enhanced chemiluminescence substrate detection system (Luminata™ Forte Western HRP Substrate, Millipore, USA). The chemiluminescent images were acquired by LAS4010 (GE Healthcare Life Sciences, USA). Densitometry was undertaken using ImageJ software.

*Data analysis*. One-way ANOVA followed by Bonferroni's post hoc test was selected as statistical analysis, and the difference between groups was considered statistically different when *p* < 0.05.

#### 2.4.4. RNA Extraction and Real-Time PCR Analysis on SIRT1

The total RNA from a portion of the left ventricular tissue was extracted using the RNeasy® Mini Kit (Qiagen, Germany), determining the purity of the RNA samples by measuring the absorbance at 260 : 280 nm. cDNA synthesis was performed with 500 ng of RNA using the iScript cDNA synthesis kit (Bio-Rad, Hercules, USA). SIRT1 and GAPDH primers were designed in intron/exon boundaries (SIRT1: For: ATGACGCTGTGGCAGATTGTT and Rev: CCGCAAGGCGAGCATAGAT; GAPDH: For: ATGTGTCCGTCGTGGATCTGAC and Rev: AGACAACCTGGTCCTCAGTGTAG) [16]. RT-PCR reactions consisted of 25 *μ*L FluoCycle® II SYBR® (EuroClone, Italy), 1.5 *μ*L of both 10 *μ*M forward and reverse primers for SIRT1 and GAPDH (Sigma-Aldrich, Italy), 3 *μ*L cDNA, and 19 *μ*L of H_2_O. All reactions were performed for 38 cycles using the following temperature profiles: 94°C for 30 s (initial denaturation); annealing temperature 90°C for 30 s (annealing); and 72°C for 1 s (extension). GAPDH was used as the housekeeping gene. mRNA levels were normalized against GAPDH mRNA levels, and relative expression was calculated by using the Ct value. PCR specificity was determined by both the melting curve analysis and gel electrophoresis.

*Data analysis*. One-way ANOVA followed by Bonferroni's post hoc test was selected as statistical analysis, and the difference between groups was considered statistically different when *p* < 0.05.

#### 2.4.5. Detection of Oxidative Stress

For histological labeling and qualitative image acquisition, left ventricles from mice were isolated and readily included in Optimal Cutting Temperature (OCT, Japan) without fixation. Cryosection 20 *μ*m thick was obtained using a manual cryostat (Leica, Germany) and collected onto glass slides. Ventricle sections were then incubated in 10 *μ*M dihydroethidium (DHE) solution at 37°C for 30 min. Sections were then rinsed 3 × 5 min in phosphate-buffered saline (PBS) and mounted for fluorescent imaging with Vectashield (Vector, Burlingame). Images were obtained for each section using a 20x objective (NA 0.50) on a Nikon Ni-E microscope (Nikon Instruments, Italy). In order to quantify ROS-positive areas in the different experimental groups, six fields of each section of the left ventricle were acquired and analyzed. Three sections for each left ventricle of five animals per group were sampled.

#### 2.4.6. Inflammatory Markers

The cardiac tissue was reduced to powder in liquid N_2_, then homogenized in PBS with an ultrasonic homogenizer (Braun, Germany) and centrifuged at 14000 rpm for 10 minutes at 4°C. The supernatant was used for the test. The concentration of total proteins in the supernatant was measured using the protein quantification assay of bicinchoninic acid (Sigma-Aldrich, USA). The concentrations of TNF-*α* and IL6 were measured in myocardial tissue using an ELISA commercial kit (R&D Systems, USA).

*Data analysis*. One-way ANOVA followed by Bonferroni's post hoc test was selected as statistical analysis, and the difference between groups was considered statistically different when *p* < 0.05.

#### 2.4.7. Histological Evaluation of Fibrosis

Histological evaluation of fibrosis was performed on 20 male mice belonging to 4 groups (5 per group): (1) three-month-old untreated, (2) six-month-old untreated, (3) twelve-month-old untreated, and (4) twelve-month-old treated. At necropsy, heart ventriculi were collected and referred to the histology laboratory. Samples were paraffin embedded and sectioned to show both ventricular walls. After rehydration of sections, Mallory trichrome staining was performed by using a commercial kit (04-020802, Mallory trichrome, Bio Optica, Italy); slides were thus dehydrated, cleared, and mounted with permanent mounting medium. Five images at 20x magnification were collected focusing at connective tissue hot spots and semiautomatically analyzed with the image processing software Nis-Elements Br (Nis-Elements Br, Nikon Instruments, Italy). Fibrosis was expressed as percent of the images occupied by the connective tissue (stained in blue with Mallory trichrome).

*Data analysis*. Values were analyzed by Bonferroni's multiple comparison test as post hoc analysis after one-way analysis of variance; significance was set at *p* < 0.05.

#### 2.4.8. Evaluation of Cardiac Tissue miR-214-3p Expression

Total microRNAs from frozen cardiac tissue were extracted and reverse transcripted by using miRNeasy Mini Kit and miScript Reverse Transcription Kit (Qiagen, Germany), respectively. Quantitative PCR was performed using the miScript SYBR-Green PCR Kit and MiScript Primer Assays specific for mmu-miR-214-3p (MIMAT0000661) and mmu-SNORD6 (Qiagen, Germany), as previously reported [33, 34]. miRNA expression was calculated using Ct method and normalized to the expression of housekeeping SNORD6.

*Data analysis*. Data were represented as the mean ± SEM. Comparisons of results were carried out by *t*-test. A *p* value < 0.05 was considered as statistically significant.

## 3. Results

### 3.1. *In Vitro* SIRT1 Enzyme Activity and *In Silico* Study of NAR as Activator of SIRT1

**Figure 1:**
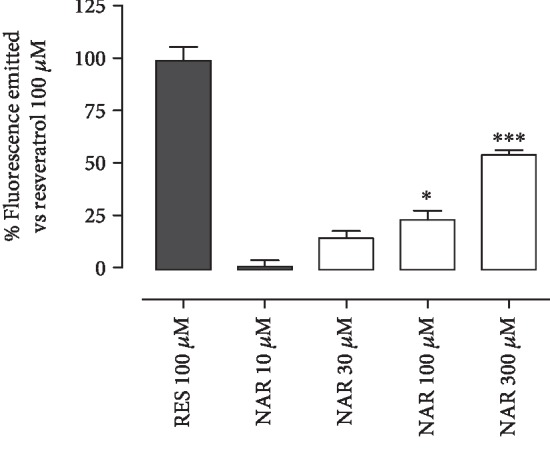
The histogram shows the % of fluorescence emitted (*vs*. RES 100 *μ*M) following the incubation of NAR 10, 30, 100, or 300 *μ*M with isolated and purified SIRT1 enzyme. The bars are expressed as average ± SEM. Statistical analysis is one-way ANOVA followed by Bonferroni's multiple comparison test (^∗^*p* < 0.05; ^∗∗∗^*p* < 0.001).

NAR was incubated with the isolated and purified SIRT1 enzyme at 10, 30, 100, and 300 *μ*M, instead RES, the reference compound, was administrated at 100 *μ*M. NAR concentration-dependently stimulated the SIRT1 enzyme activity, leading to a significant activation of 24 ± 4% at 100 *μ*M and to 55 ± 2% at 300 *μ*M (Figure 1).

**Figure 2:**
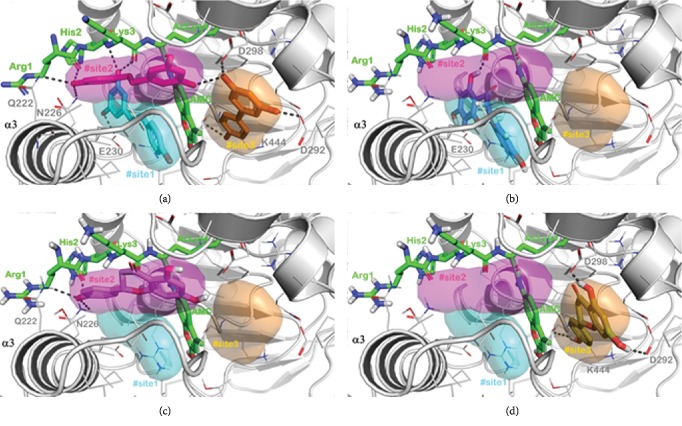
(a) SIRT1 (light grey cartoon) cocrystallized with RES (sticks representation) (PDB ID: 5BTR). In the picture was highlighted the three different binding sites of RES (#site1 is represented in cyan; #site2 is represented in magenta, while #site3 is represented in orange). The significant residues in the binding sites are represented by lines. (b) Putative binding mode of NAR (represented by light blue sticks) into #site1. The interacting residues of the binding site are labeled. (c) Putative binding mode of NAR (represented by light blue sticks) into #site2. The interacting residues of the binding site are labeled. (d) Putative binding mode of NAR (represented by light blue sticks) into #site3. The binding site interacting residues are labeled. The acetylated p53-AMC peptide is represented by green sticks. The H-bonds are represented by dark grey dotted lines. The pictures were generated by PyMOL (The PyMOL Molecular Graphics System, v1.8; Schrödinger, LLC, New York, 2015).

In order to gain insight into the interaction of NAR within the enzyme SIRT1 at molecular level, we performed a computational investigation based on the application of a molecular docking protocol. This calculation was done as described in Materials and Methods. In particular, due to the high similarity of NAR with RES, it is plausible to hypothesize that the mentioned molecules could share the same binding mode. The results in Figure 1 showed that NAR behaves as activator of SIRT1 as observed for RES. Concisely, RES is able to activate SIRT1 by interacting with a specific region of the N-terminal domain (NTD) of SIRT1. In particular, as depicted in Figure 2(a), the crystal structure revealed that three molecules of RES are able to bind the mentioned domain in three distinct regions of the NTD [23], namely, #site1, #site2, and #site3. In addition, the experimental structure of RES in complex with SIRT1 was obtained with a peptide sequence (7-amino-4-methylcoumarin AMC-containing peptide), representing the p53-interacting residues and located at the interface between NTD and catalytic domain (CD) (Figure 2(a)). The abovementioned binding sites were found to differently contribute to the activation of SIRT1. Two molecules of RES mediate the interaction between the AMC peptide and the NTD of SIRT1 and are principally responsible for promoting tighter binding between SIRT1 and the peptide and consequently the stimulation of SIRT1 activity. Notably, this kind of condition is the same with that used in the assay for assessing the activation of SIRT1 as reported in the previous paragraph. As experimentally demonstrated, NAR and RES act as SIRT1 activators with different potency. So, due to the high similarity between the molecules, by a computational analysis, it is possible to rationalize this phenomenon. We independently docked NAR and RES at the three binding sites for evaluating the binding affinity. Starting from the #site1, we observed that NAR and RES established a series of contacts with the enzyme (H-bonds with E230) and also with the AMC peptide (H-bonds with Lys3 and hydrophobic interactions with AMC moiety) (Figures 2(a) and 2(b)). We found a binding affinity of -6.132 kcal/mol for NAR and -7.127 kcal/mol for RES which was observed to establish more relevant hydrophobic contacts within the #site1.

Regarding the #site2, NAR targets key residues in activating SIRT1 (Q222 and N226) (Figure 2(c)). An H-bond was observed with the backbone of Arg1 of the AMC peptide. NAR showed a binding affinity for this site of -6.146 kcal/mol while RES of -7.159 kcal/mol. The above described binding sites were found extremely relevant in activating SIRT1, while the third site is defined as “accessory site,” showing a poorer contribution in activating SIRT1 [23]. In this binding site, the affinity found for NAR is -6.179 kcal/mol while for RES it is -7.204 kcal/mol. In summary, based on the computational investigation, the before binding mode of NAR could be the same described for RES. In fact, significant affinities were found for NAR towards the three binding sites, suggesting that the compound can bind the same binding sites of RES. The latter is relevant for promoting tighter binding between SIRT1 and the peptide and consequently for the activation of SIRT1 [23]. Moreover, the slight difference in binding affinities previously discussed could rationally explain the slight reduction, found for NAR, in activating SIRT1 with respect to RES.

### 3.2. Antisenescence Effects of SIRT1 Mediated on H9c2 Cells

**Figure 3:**
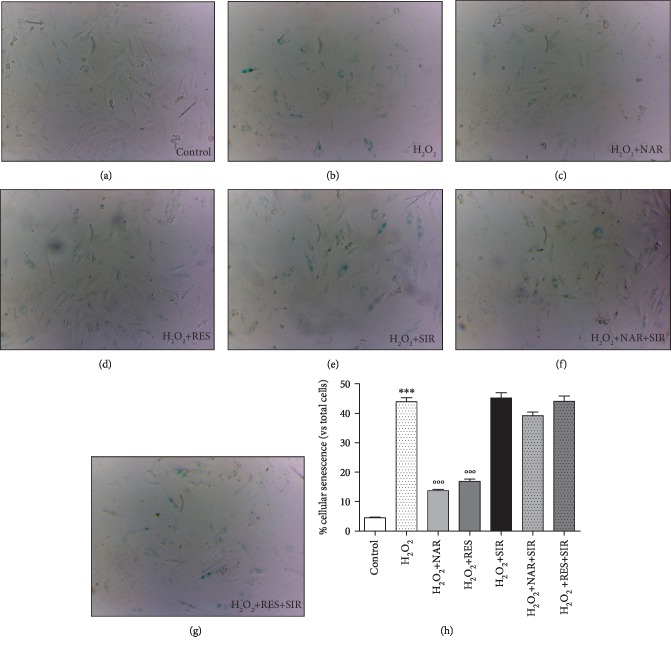
Effects of NAR on H9c2 senescence-associated *β*-galactosidase staining. Representative phase-contrast photomicrographs of control cells (a), H_2_O_2_-injured cells (b), H_2_O_2_-injured cells treated with NAR 40 *μ*M (c) or RES 25 *μ*M (d) or SIR 20 *μ*M (e), and H_2_O_2_-injured cells treated with NAR plus SIR (f) or RES plus SIR (g). (h) Histogram shows the percentage of cellular senescence. Data are shown as the percentages of *β*-galactosidase-positive cells with respect to the total cell number of the sample. Each bar represents the mean ± SEM. Statistical analysis is one-way ANOVA followed by Bonferroni's multiple comparison test (^∗∗∗^*p* < 0.001). ^∗^Indicates the significance versus control; °indicates the significance versus H_2_O_2_-injured cells.

The sa-*β*-Gal staining highlighted that H_2_O_2_ 60 *μ*M was able to significantly induce cellular senescence (Figure 3). Both NAR 40 *μ*M (Figures 3(c) and 3(h)) and RES 25 *μ*M (Figures 3(d) and 3(h)) markedly protected H9c2 from cell senescence. As expected, the treatment with the acronimous SIR (20 *μ*M) evidenced a robust sa-*β*-Gal staining in injured cells (Figure 3(e)). Notably, the cotreatments with NAR plus SIR and RES plus SIR caused the complete loss of antisenescence properties of polyphenols (Figures 3(f)–3(h)).

### 3.3. Cardiometabolic Parameters

Cholesterol, LDL, and non-LDL levels did not show significant changes in the different ages (3, 6, and 12 months); on the other hand, a significant increase of triglyceride levels and a marked reduction of HDL levels were observed in 12-month-old mice.

NAR treatment was not associated with any variation in food and water intake if compared with the vehicle group (data not shown).

**Figure 4:**
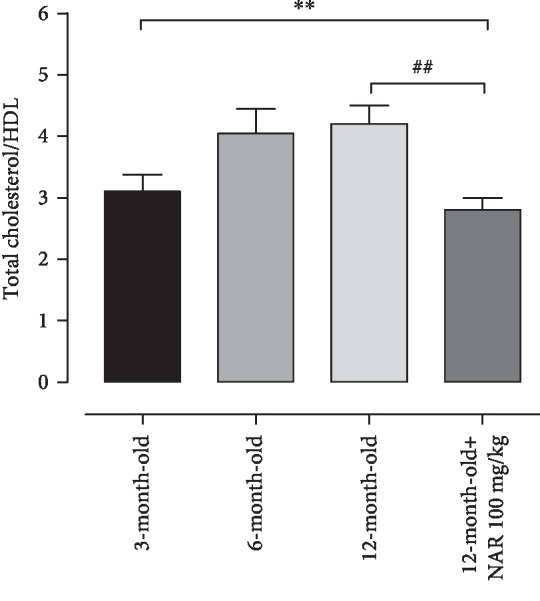
The histogram shows cardiovascular risk (measured as ratio between cholesterol and HDL levels) in 3-, 6-, and 12-month-old mice and in 12-month-old mice treated with NAR 100 mg/kg. The bars are expressed as average ± SEM. Statistical analysis is one-way ANOVA followed by Bonferroni's multiple comparison test (^∗∗^*p* < 0.01). The number of animals for each treatment group is *n* = 15. ^∗^Indicates the significance versus 3-month-old mice; ^#^indicates the significance versus 12-month-old mice.

Interestingly, the daily treatment with NAR markedly reduced triglycerides and increased HDL levels in 12-month-old mice (Table 1). Overall, cardiovascular risk of old mice was significantly lower in NAR-treated animals and it was similar to that of young controls (Figure 4).

No significant changes were observed on glycemic parameters (glycemia and glycated haemoglobin, Table 1).

### 3.4. SIRT1 Expression

**Figure 5:**
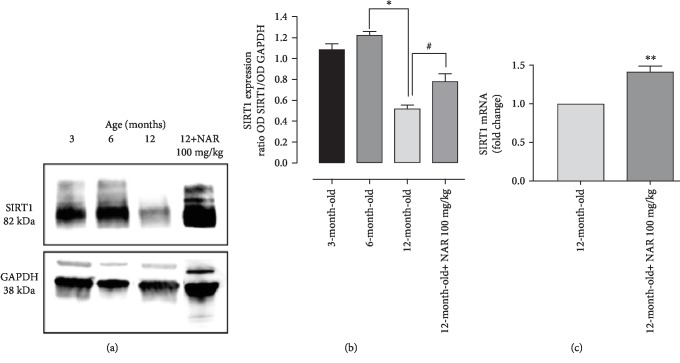
The protein levels of SIRT1 undergo a reduction as a function of increasing age. (a) An example of western blot where a reduction of SIRT1 levels is visible. At 12 months of age, SIRT1 expression levels are significantly lower than those of 3 and 6 months of age. Conversely, the chronic treatment for 6 months with NAR effectively increases the SIRT1 protein levels, compared to mice of the same age treated with the vehicle alone. (b) The histogram shows the quantitative analysis executed by densitometry. (c) Relative expression of mRNA SIRT1 normalized *vs*. the housekeeping gene GAPDH in cardiac tissue from mice fed with NAR (100 mg/kg/day) or its vehicle, for 6 months. The bars are expressed as average ± SEM. Statistical analysis is one-way ANOVA followed by Bonferroni's multiple comparison test (^∗^*p* < 0.05; ^∗∗^*p* < 0.01). The number of animals for each treatment group is *n* = 5. ^∗^Indicates the significance versus 6-month-old mice; ^#^indicates the significance versus 12-month-old mice.

Myocardial SIRT1 expression showed an age-related decrease; in particular, no differences between young (3-month-old) and young-adult (6-month-old) mice were shown but a drastic and significant reduction of SIRT1 expression (about half) was observed in 12-month-old mice. Conversely, 12-month-old mice treated with NAR showed a significant increase of SIRT1 levels in cardiac tissue, when compared with the vehicle-treated mice of the same age. In agreement with the protein levels, a significant increase of mRNA expression has been also observed in NAR-treated 12-month-old mice if compared with the corresponding controls (Figures 5(a)–5(c)).

### 3.5. Reactive Oxygen Species Production

**Figure 6:**
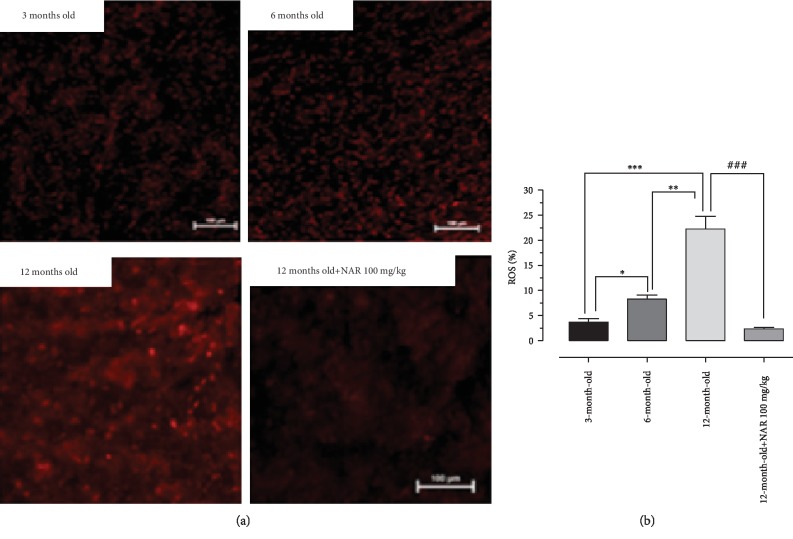
ROS levels in the cellular environment increase proportionally with age. (a) The images show that an increase in DHE-positive nuclei is obtained with the increase of the age, indicating a higher presence of ROS in the cellular environment, which converts dihydroethidium into ethidium (bright red nuclei). The treatment with NAR is effective in reducing the number of DHE-positive cells, indicating a lower concentration of ROS in the cellular environment. (b) The histogram shows the quantification of ROS (%). In animals treated with NAR, ROS levels are significantly reduced compared to the group of animals treated with vehicle alone. The bars are expressed as average ± SEM. Statistical analysis is one-way ANOVA followed by Bonferroni's multiple comparison test (^∗^*p* < 0.05; ^∗∗^*p* < 0.01; ^∗∗∗^*p* < 0.001). The number of animals for each treatment group is *n* = 5. ^∗^Indicates the significance versus 3-month-old mice; ^#^indicates the significance versus 12-month-old mice.

DHE staining highlighted an age-dependent production of ROS. Indeed, in the hearts from young mice, the DHE-positive areas were minimal, whereas in 12-month-old mice ROS levels were markedly higher than young controls. Conversely, in animals treated with NAR for six months, DHE-positive areas were significantly reduced, showing values superimposable with the young controls (Figures 6(a) and 6(b)).

### 3.6. Inflammatory Markers

**Figure 7:**
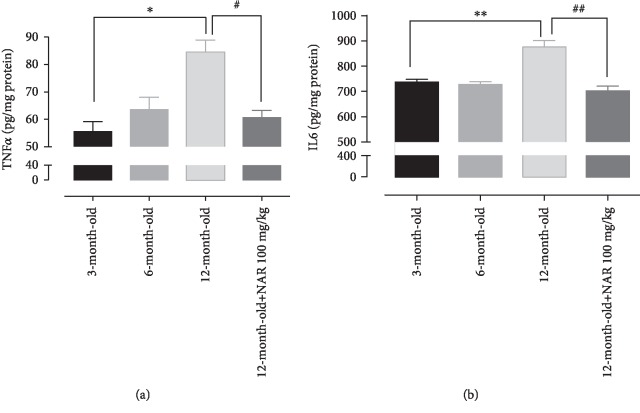
The histograms show (a) the TNF-*α* levels and (b) the IL6 levels measured in 3-, 6-, and 12-month-old mice and 12-month-old treated with NAR 100 mg/kg mice. Statistical analysis is one-way ANOVA followed by Bonferroni's multiple comparison test (^∗^*p* < 0.05; ^∗∗^*p* < 0.01). The number of animals for each treatment group is *n* = 5. ^∗^Indicates the significance versus 3-month-old mice; ^#^indicates the significance versus 12-month-old mice.

In agreement with the literature, cardiac levels of two well-known inflammatory markers (TNF-*α* and IL6) showed an age-dependent increase, thus indicating a typical chronic low-grade inflammation in senescent tissue; in particular, both TNF-*α* and IL6 levels were significantly higher in 12-month-old mice than in young controls. Interestingly, the hearts of mice treated with NAR had TNF-*α* and IL6 levels markedly reduced and similar to those of 3-month-old mice (Figures 7(a) and 7(b)).

### 3.7. Basal Mitochondrial Membrane Potential

**Figure 8:**
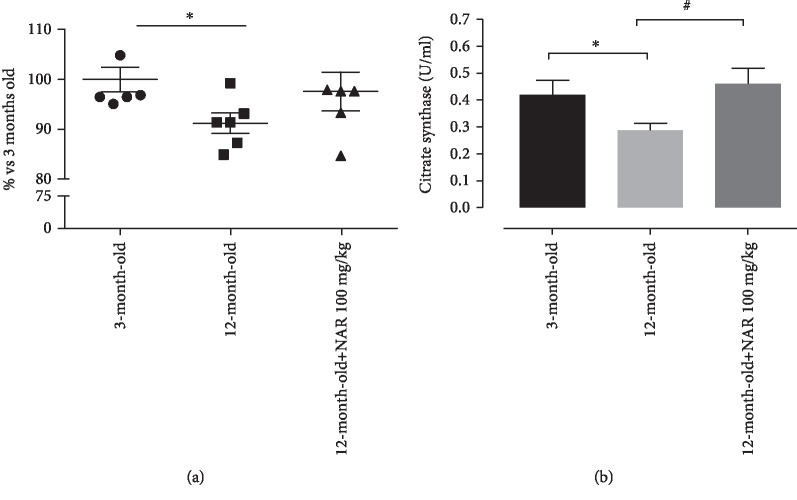
(a) Change % of the mitochondrial membrane potential observed in 12-month-old mice and in 12-month-old mice treated with NAR 100 mg/kg vs. the young group. The number of animals for each treatment group is *n* = 6. (b) Citrate synthase activity (U/mL) of 3-month-old mice, 12-month-old mice, and 12-month-old mice treated with NAR 100 mg/kg. The number of animals for each treatment group is *n* = 8. ^∗^Indicates the significance versus 3-month-old mice; ^#^indicates the significance versus 12-month-old mice. Statistical analysis is one-way ANOVA followed by Bonferroni's multiple comparison test (^∗^*p* < 0.05).

In cardiac isolated mitochondria, a significant depolarization of mitochondrial potential membrane (ΔΨ) was recorded in 12-month-old animals (about 9%) *vs*. the young controls. A trend of increase was observed in cardiac mitochondria isolated from mice treated with NAR (Figure 8(a)).

### 3.8. Citrate Synthase Activity

Citrate synthase (CS) activity reflects metabolic condition of cardiac tissue and, in our experimental conditions, was 0.42 ± 0.054 U/mL in 3-month-old mice. In cardiac mitochondria isolated from 12-month-old mice, the CS activity was significantly lower (0.29 ± 0.026 U/mL) than that in young controls; on the other side, mitochondria from NAR-treated mice of the same age showed a significantly higher CS activity (0.46 ± 0.058 U/mL; Figure 8(b)).

### 3.9. miR-214-3p Expression

**Figure 9:**
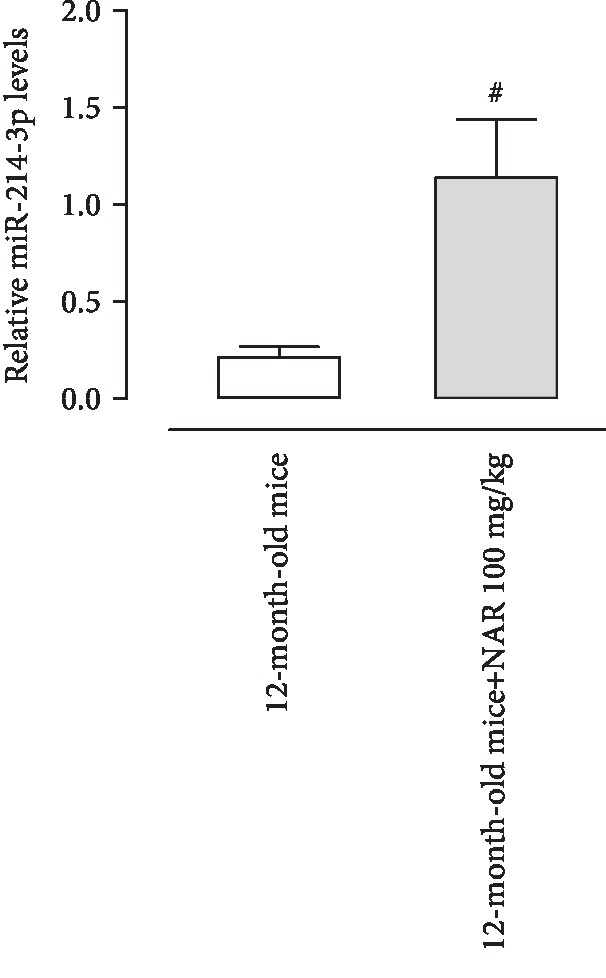
Relative expression of miR-214-3p normalized vs. the housekeeping gene SNORD6 in cardiac tissue from mice fed with NAR (100 mg/kg/day) or its vehicle, for 6 months. All data sets were represented as the mean ± standard error of mean (SEM). Comparisons of results between two groups were carried out by *t*-test. A *p* value < 0.05 was considered as statistically significant.

In cardiac tissue of 12-month-old mice treated with NAR for 6 months, miR-214-3p expression was significantly upregulated (about 7-fold), if compared to levels of mice treated with vehicle for the same period (Figure 9).

### 3.10. Histological Evaluation of Fibrosis

**Figure 10:**
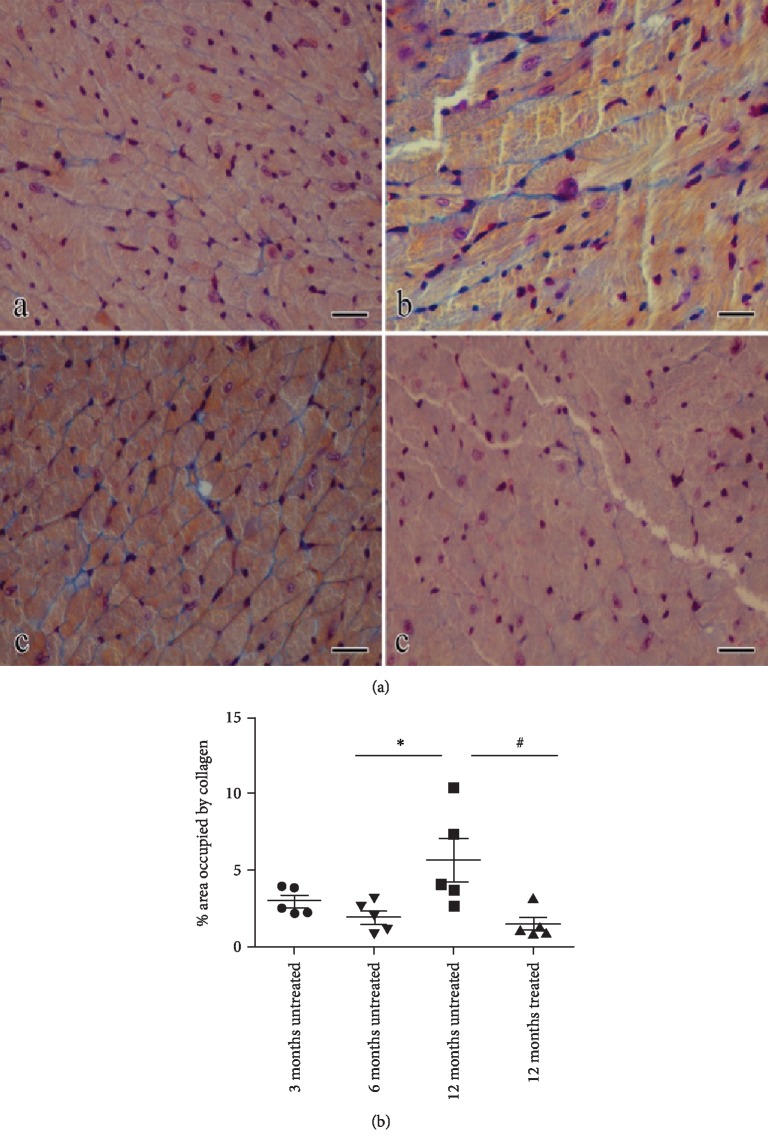
(a) Mallory trichrome-stained section of heart ventriculi showed in 3-, 6-, and 12-month-old mice and in 12-month-old mice treated with NAR 100 mg/kg. (b) Quantitative analysis of the age-related fibrotic areas. Statistical analysis is one-way ANOVA followed by Bonferroni's multiple comparison test (^∗^*p* < 0.05; ^∗∗^*p* < 0.01; ^∗∗∗^*p* < 0.001). The number of animals for each treatment group is *n* = 5. ^∗^Indicates the significance versus 6-month-old mice; ^#^indicates the significance versus 12-month-old mice.

Histological examination of Mallory trichrome-stained section of heart ventriculi showed that myocardial fibrosis increases with age (3 months old: 2.9 ± 0.9%; 6 months old: 1.9 ± 0.9%; 12 months old: 5.6 ± 3.2%). Specifically, differences were statistically significant between 6-month-old mice and 12-month-old mice. NAR treatment markedly reversed such an age-dependent fibrotic degeneration (12 months old *plus* NAR: 1.5 ± 0.9%); indeed, collagen deposition in the myocardium of 12-month-old NAR-treated mice was statistically different from 12-month-old untreated animals (Figure 10).

## 4. Discussion

Ageing mediates various structural and functional alterations in the cardiovascular system. In particular, fibrotic processes, characterized by the accumulation of collagen type 1 and amyloid, occur in the myocardium during normal ageing and lead to myocardial hypertrophy and severe cardiovascular diseases, such as chronic ischemia, cardiomyopathies, and heart failure [35, 36]. It is also well-known that during ageing, a chronic, sterile, and low-grade inflammation, called inflammaging, develops and contributes to the pathogenesis of age-related diseases [3]. Moreover, SIRT1 levels and activity decrease with age [9]; hence, considering its key role in the production of antioxidant defenses, ageing is unavoidably accompanied with oxidative stress. Moreover, ageing-associated reduction of SIRT1 leads to the loss of control on acetylation of target proteins including p53, Nrf2, and FOXO3, thereby enhancing inflammatory, prosenescent, and apoptotic responses [37].

In this work, no difference in cardiac SIRT1 expression has been found between 3-month-old and 6-month-old mice, whereas a significant reduction has been observed in 12-month-old mice; indeed, according to the literature [9], SIRT1 expression is about halved in 12-month-old animals.

Although murine models are widely used in preclinical studies to evaluate the human physiology and its modulation, a number of differences in the developmental duration and phases between mice and human have been revealed. Therefore, a precise correlation between mice age and human age needs to consider various aspects of the life stages (not only lifespan but also reproductive age, puberty age, and weaning period). Indeed, all the proposed methods are not exactly adequate, and often, more than one method is used. Probably, the most precise approach for correlating the age of the mouse with the age of human is to consider the expression of specific markers of senescence [38].

At this regard, we observed a marked reduction of cardiac SIRT1 expression in 12-month-old mice, thus confirming that the myocardium of this animal group is in a senescence stage. Moreover, as already published [39], we observed in this animal group a higher cardiovascular risk and triglyceride levels significantly increased if compared with those of 3- and 6-month-old mice.

Furthermore, an age-dependent production of ROS has been observed by means of DHE staining; in particular, extensive positive areas are present in cardiac tissue of 12-month-old mice, according to the reduction of SIRT1 levels and, subsequently, of Nrf2-mediated antioxidant defenses. Moreover, a significant increase of the inflammatory markers IL6 and TNF-*α* has been observed between 3- and 12-month-old mice.

Finally, the strongest evidence of the myocardial senescence is the fibrotic process. We did not observe changes between 3- and 6-month-old mice, but a surge of collagen accumulation has been highlighted in 12-month-old mice; indeed, an extended area occupied by collagen (about 6%) has been observed, although this value seems to be particularly variable in relation with individual factors.

Hoppel et al. reported that cardiac mitochondria undergo detrimental changes during the ageing processes [40]. Mitochondria participate in cell bioenergetics and metabolism; therefore, they play a critical role in lifespan determination and ageing. The best-known hypothesis to explain ageing is the free radical theory, and ROS are mainly generated at the level of the mitochondrial respiratory chain. Hence, dysfunctional mitochondria produce less ATP and it compromises different cellular features [41]. In this context, the most reliable indicator of mitochondrial efficiency is the membrane potential value that is about -180 mV in well-energizing organelles. However, a mild significant depolarization of ΔΨ is common in mitochondria from elderly individuals; indeed, although the physiological functions are guaranteed, there is a lower tolerance toward several types of insults in senescent myocardium, such as myocardial infarct and heart failure [42]. At this regard, we observe a partial depolarization (about 9%) in mitochondria obtained from 12-month-old mice. This result has been also reported by other researchers, both at cardiac level and in other organs [43].

Citrate synthase is a mitochondria matrix enzyme that acts as a rate-limiting enzyme in the first step of the Krebs cycle. It is extensively used as a metabolic marker for evaluating mitochondrial oxidative capacity [44]. The hearts of 12-month-old mice present a partially compromised activity of the citrate synthase enzyme (of about 30%). Taken together, this experimental evidence demonstrates that the myocardium from 12-month-old mice is in a senescent state and confirms the reliability of this animal model.

Previous papers of ours reported multiple beneficial effects of the Citrus flavonoid NAR at the cardiovascular level. We demonstrated vasorelaxant and cardioprotective properties mediated through the activation of large conductance calcium-activated potassium channels (BKCa) that are described both at sarcolemmal and, more recently, at mitochondrial level [11, 12]. Moreover, unlike the usual cardioprotective agents (i.e., diazoxide), NAR guarantees myocardial protection also in mature animals (12-month-old), suggesting other possible mechanisms involved in its pharmacological profile [13]. At this regard, Da Pozzo and her colleagues demonstrated, for the first time, antiageing effects of NAR in *in vitro* model, in which senescence has been induced through a treatment with H_2_O_2_ [16].

Interestingly, in the present study, we observed that NAR activates the isolated SIRT1 enzyme similarly to the reference activator RES; furthermore, the interaction between NAR and SIRT1 has been also highlighted by the computational study. Moreover, in an *in vitro* model of H_2_O_2_-induced cell senescence, NAR, like RES, showed antisenescence effects in rat cardiomyoblasts. Interestingly, this effect has been completely abolished in cells cotreated with the well-known SIRT1 antagonist SIR, supporting the hypothesis of an involvement, at least in these experimental conditions, of SIRT1 in the molecular mechanism of NAR. Furthermore, a significant increase of SIRT1 expression in the myocardium of animals treated for 6 months with the Citrus flavonoid has been also observed.

It is well-known that RES, through the activation of SIRT1, is able to trigger intracellular downstream events that contribute to the reinforcement of antioxidant defenses and the mitigation of inflammatory processes [45, 46]. Similarly, NAR markedly reduces cardiac oxidative stress in 12-month-old mice, restoring the tissue oxidative state to that of the young control; moreover, NAR restrains the production of inflammatory markers (IL6 and TNF-*α*). These results find a confirmation in our very recent paper, in which a supplemental diet with NAR (100 mg/kg for 6 months) effectively slowed down oxidative stress associated with retinal degeneration on rd10 mice [25].

Noteworthly, we observed also an upregulation of miR-214 in cardiac tissue after NAR treatment. miR-214 is reported to have cardioprotective activity preventing cell death and oxidative stress [47, 48]. Moreover, miR-214 has been also reported to be relevant in age-related diseases, such as Parkinson; indeed, Wang and colleagues have demonstrated an overexpression of miR-214 in Parkinson disease mice treated with RES [49].

Furthermore, levels of miR-214 are decreased in senescent endothelial cells [50] and another flavonoid compound, luteolin, is able to increase the levels of miR-214 in mesenteric arterioles of mice [37]. Intriguingly, miR-214 is also a regulator of the transcriptional repressor *Hypermethylated In Cancer 1* (HIC1), a predicted target (TargetScanMouse 7.1) of mmu-miR-214-3p, and also a negative regulator of SIRT1 since it forms a transcriptional repression complex with SIRT1 deacetylase, and this complex directly binds the SIRT1 promoter repressing its transcription [51]. Although further specific investigations will be aimed at addressing the miR-214/HIC1/SIRT1 pathway, these preliminary data strengthen the protective role of NAR.

Furthermore, cardiovascular risk and triglyceride levels are significantly reduced in animals treated with NAR when compared with animals of the same age. Indeed, it is well-known that age is a main physiological factor that has a strong influence on plasma levels of lipids, and although the cardiovascular risk is considered a general parameter indicative of metabolic profile, commonly, it is associated with a lower tolerance of the myocardium towards cardiovascular insult. Therefore, these results contribute to strengthen the belief that the Citrus flavanone is endowed with cardioprotective properties.

NAR improves the ΔΨ value and restores the activity of citrate synthase enzyme, similarly to those of young animals, suggesting that it is endowed with beneficial effects on the mitochondrial efficiency and on mitochondrial oxidative capacity.

Further evidence of the antiageing effects of NAR is obtained by histomorphological analysis; strikingly, NAR treatment limits the fibrotic areas in the myocardium of 12-month-old mice, where the percentage of collagen deposits is similar to that of 3-month-old mice.

## 5. Conclusions

We demonstrate that NAR, at least in part *via*, modulation of SIRT1 enzyme, can slow down the myocardial degradative processes associated with senescence; and taken together, these results suggest that a chronic treatment with the Citrus flavonoid NAR could guarantee beneficial effects at the myocardial level also in humans. A limitation of this study is represented by the inability to correlate with absolute certainty the antiageing effects observed *in vivo* with the presence of NAR at systemic level. Indeed, on the basis of the results on the isolated enzyme and on H9c2 cells, we can assert that SIRT1 is the target of the ageing-preventive effects of NAR; however, since NAR is converted in several metabolites *in vivo*, we cannot exclude the possibility that, at least in part, the antisenescence profile observed in mice is due to some active metabolites. In fact, it is well-known that several NAR metabolites are biologically active, in particular, those deriving from hydroxylation, methylation, or dehydrogenation, among which hesperetin, apigenin, and eriodictyol [52]. Moreover, a contribution of intestinal microbiota cannot be excluded, considering that an amount of short-chain fatty acids (SCFAs), propionic and acetic acid derivatives, have been described as metabolism end-products of polyphenols, including Citrus flavonoids [53]. Furthermore, an interesting role of SCFAs in the beneficial effects of flavonoids is emerging; in particular, they can contribute to the eubiosis condition and to regulate metabolic processes, also improving the mitochondrial function [54]. Therefore, future experiments will be certainly directed to examine such aspect.

## Figures and Tables

**Table 1:** Cardiometabolic parameters measured in the blood. In the table, the cholesterol, LDL, HDL, triglyceride, glycemia, and glycated haemoglobin levels are reported. The values are expressed as average ± SEM. The number of animals for each treatment group is *n* = 15. Statistical analysis is one-way ANOVA followed by Bonferroni's multiple comparison test (^∗^*p* < 0.05; ^∗∗^*p* < 0.01). ^∗^Indicates significance vs. 3-month-old, ^#^indicates significance vs. 12-month-old.

	3-month-old	6-month-old	12-month-old	12-month-old+NAR 100 mg/kg
Total cholesterol (mg/dL)	76 ± 6	70 ± 6	71 ± 3	72 ± 3
Triglycerides (mg/dL)	59 ± 3	66 ± 1	95±15^∗∗^	70 ± 5^##^
HDL (mg/dL)	26 ± 2	19 ± 4	17±1^∗∗^	26 ± 3^#^
LDL (mg/dL)	39 ± 5	38 ± 4	35 ± 3	32 ± 2
Glycemia (mg/dL)	87 ± 14	85 ± 4	103 ± 9	94 ± 7
HbA1C (mmol/mol)	29 ± 1	30 ± 1	29 ± 1	30 ± 1

## Data Availability

The data used to support the findings of this study are available from the corresponding author upon request.

## Supplementary Materials

Supplementary Materials Supplement Materials 1: cumulative curve obtained with resveratrol on in vitro spectrofluorimetric assay. Supplement Materials 2: time-dependent decrease of SIRT1 expression obtained from the hearts of 3, 6, 9, and 12-month-old mice.
