# Supplementary material for: The Citrus Flavonoid Naringenin Protects the Myocardium from Ageing-Dependent Dysfunction: Potential Role of SIRT1
Source: Oxid Med Cell Longev. 2020 Jan 25;2020:4650207. doi: 10.1155/2020/4650207 (PMC7003265; doi:10.1155/2020/4650207)
Supplement: Supplementary Materials — Supplement Materials 1: cumulative curve obtained with resveratrol on in vitro spectrofluorimetric assay. Supplement Materials 2: time-dependent decrease of SIRT1 expression obtained from the hearts of 3, 6, 9, and 12-month-old mice. [file 4650207.f1.pptx]

## Slide 1
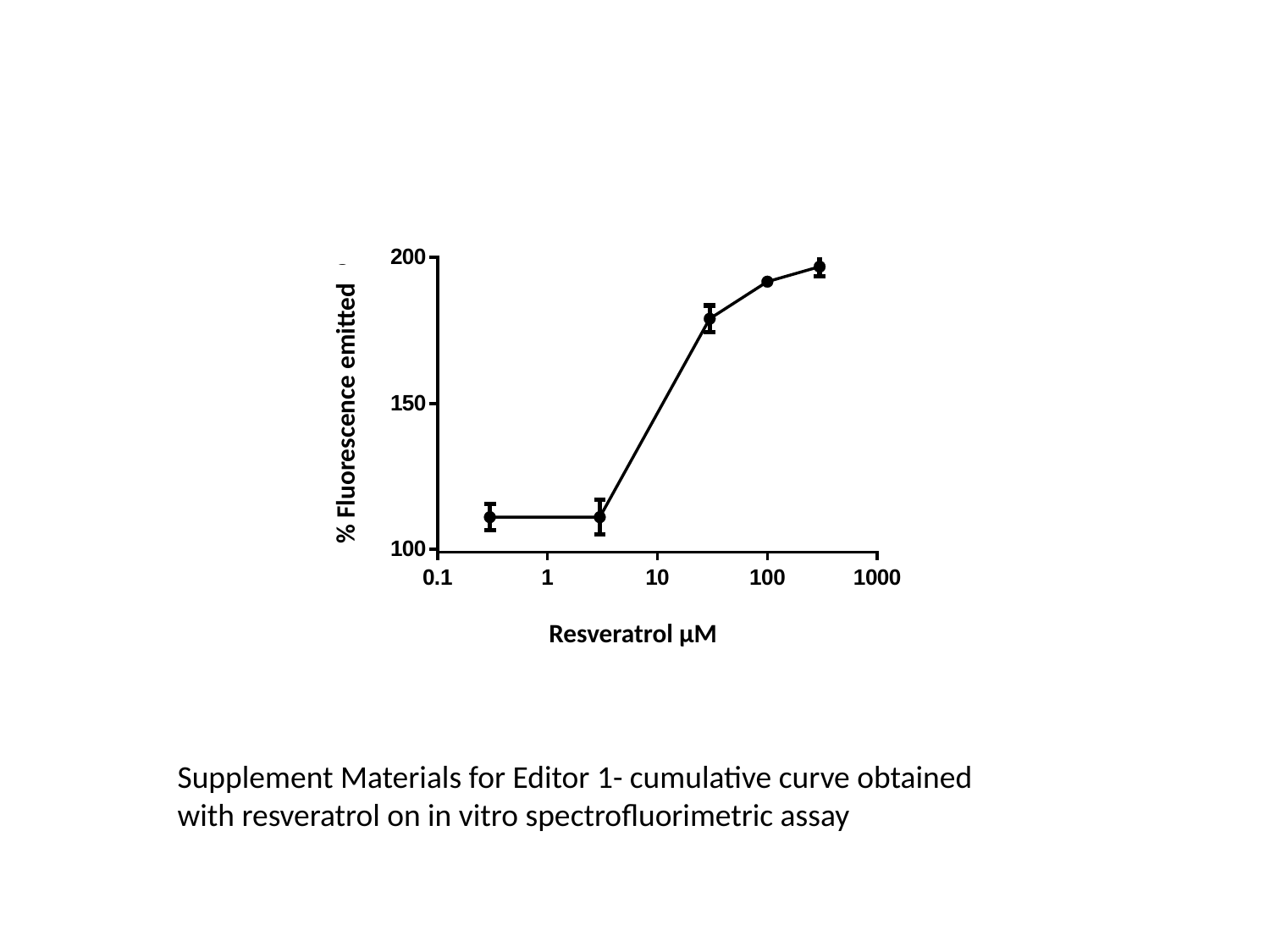

% Fluorescence emitted
Resveratrol µM
Supplement Materials for Editor 1- cumulative curve obtained with resveratrol on in vitro spectrofluorimetric assay

## Slide 2
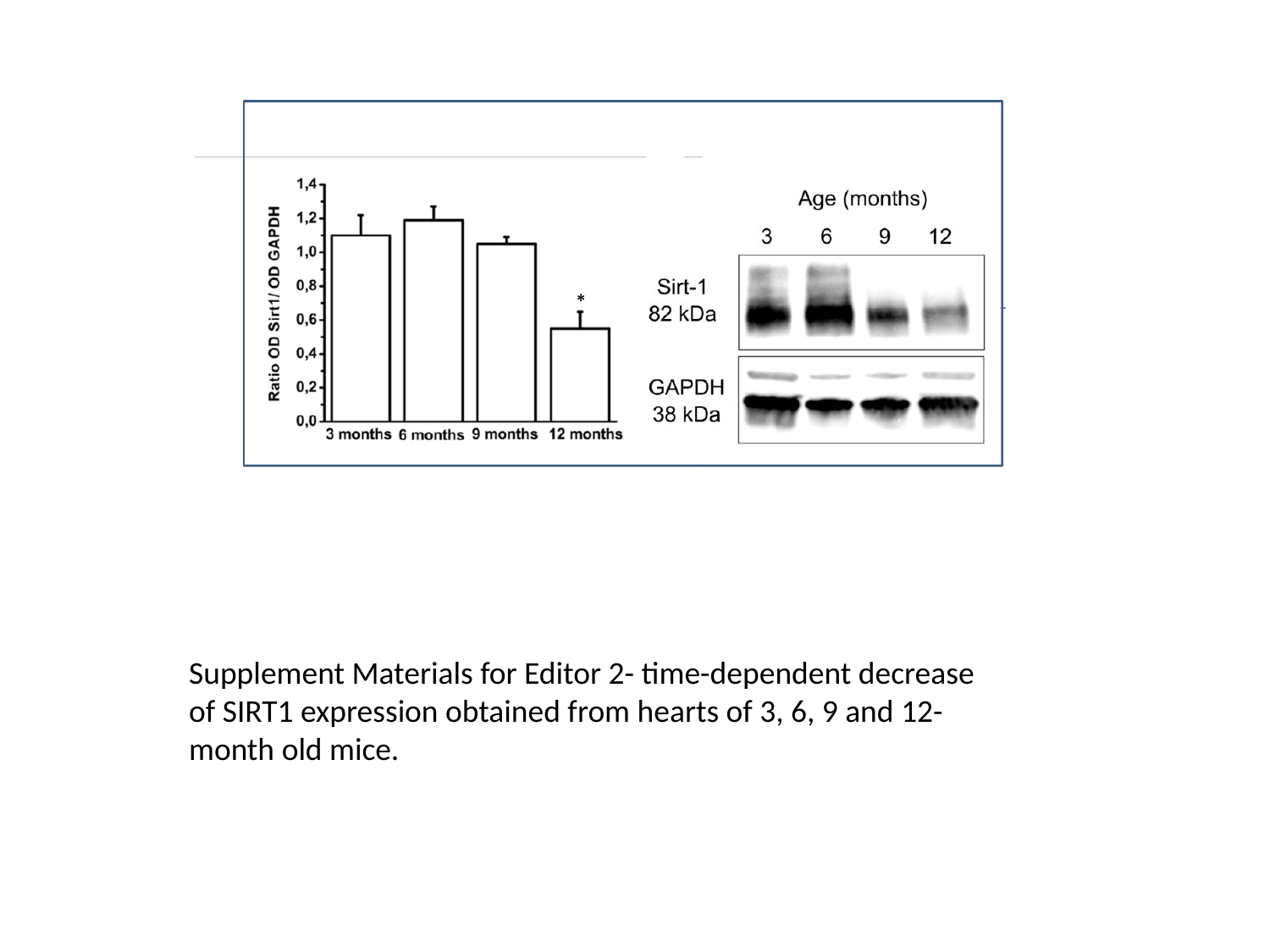

GROUP 1 GROUP 2
Supplement Materials for Editor 2- time-dependent decrease of SIRT1 expression obtained from hearts of 3, 6, 9 and 12-month old mice.
